# Paternal Finasteride Treatment Can Influence the Testicular Transcriptome Profile of Male Offspring—Preliminary Study

**DOI:** 10.3390/cimb43020062

**Published:** 2021-07-31

**Authors:** Agnieszka Kolasa, Dorota Rogińska, Sylwia Rzeszotek, Bogusław Machaliński, Barbara Wiszniewska

**Affiliations:** 1 Department of Histology and Embryology, Pomeranian Medical University (PMU), Powstańców Wlkp. 72 Avene, 70-111 Szczecin, Poland; sylwia.rzeszotek@pum.edu.pl (S.R.); barbara.wiszniewska@pum.edu.pl (B.W.); 2 Department of General Pathology, Pomeranian Medical University, Powstańców Wlkp. 72 Avene, 70-111 Szczecin, Poland; doroginska@gmail.com (D.R.); machalin@pum.edu.pl (B.M.)

**Keywords:** testis, transcriptome, microarrays analysis, finasteride, DHT deficiency

## Abstract

(1) Background: Hormone-dependent events that occur throughout spermatogenesis during postnatal testis maturation are significant for adult male fertility. Any disturbances in the T/DHT ratio in male progeny born from females fertilized by finasteride-treated male rats (F0:Fin) can result in the impairment of testicular physiology. The goal of this work was to profile the testicular transcriptome in the male filial generation (F1:Fin) from paternal F0:Fin rats. (2) Methods: The subject material for the study were testis from immature and mature male rats born from females fertilized by finasteride-treated rats. Testicular tissues from the offspring were used in microarray analyses. (3) Results: The top 10 genes having the highest and lowest fold change values were mainly those that encoded odoriferous (*Olfr*: *31*, *331*, *365*, *633*, *774*, *814*, *890*, *935*, *1109*, *1112*, *1173*, *1251*, *1259*, *1253*, *1383*) and vomeronasal (*Vmn1r*: *50*, *103*, *210*, *211*; *Vmn2r*: *3*, *23*, *99*) receptors and *RIKEN cDNA 5430402E10*, also known as odorant-binding protein. (4) Conclusions: Finasteride treatment of male adult rats may cause changes in the testicular transcriptome of their male offspring, leading to a defective function of spermatozoa in response to odorant-like signals, which are recently more and more often noticed as significant players in male fertility.

## 1. Introduction

Without a doubt, the morphology and function of the male gonad are controlled by the hypothalamic–pituitary–gonad axis and locally produced androgens—testosterone (T) and more biologically active dihydrotestosterone (DHT) [1]. Transformation of T into DHT is catalyzed by 5a-reductase (5α-red) [2]. It was shown that in the male reproductive tract, the most common isoform of this enzyme is 5α-reductase type 2 (5α-red2) [3]. Inhibition of this enzyme by finasteride [4] has found application in clinical practice—in the therapy of prostate cancer, benign prostatic hyperplasia (BPH), and androgenetic alopecia (AGA; prematurely balding young men) [5,6]. In the 1990s, finasteride was introduced to medical care and was considered a safe drug, without any “*side effects*” on male fertility, and this view is still maintained among dermatologists [7,8]. However, there are many studies documenting contrary findings [9,10], a decrease in semen parameters [5,11,12], difficulties in fertilization [5], psychological problems such as reduced libido, orgasms, and erectile disorders [13], melancholy, and even suicidal thoughts [14]. Much more sensitive analysis has shown elevated sperm DNA fragmentation in patients treated with low doses of finasteride [15,16]. Studies with transmission electron microscopy revealed an altered sperm morphology resembling necrosis, while FISH (fluorescent in situ hybridization) data showed elevated frequencies of diploidy and sex chromosome disomy [17]. One year after the cessation of finasteride treatment, the meiotic pattern did not change, and elevated diploidy and sex chromosome disomy were still present; however, motility and sperm morphology were improved [17]. This so-called post-finasteride syndrome (PFS) was recently added to the list of genetic and rare diseases by the United States National Institutes of Health (NIH) [18,19].

In utero, dose-dependent exposure to finasteride induced permanent effects on androgen-sensitive end points, such as anogenital distance, nipple retention, and malformations of the male rat and rhesus monkey reproductive tracts (absence of prostate lobes, ectopic testes, small scrotum, small penis, hypospadias) [20,21]. Initially, it was suggested that there was still no documented full-term pregnancy or live birth from couples in which the man had received finasteride [15], so caution has been advised regarding the use of finasteride by male members of couples who are planning to have children [22]. Probably for these reasons, in 2015 in South Korea, a clinical study was conducted which proved that in 19 couples with documented paternal exposure to finasteride, 13 couples (68.4%) gave birth to normal full-term babies, and six cases (31.6%) resulted in spontaneous (*n* = 3) or artificial (*n* = 3) abortions [23]. In our previous study [24], we observed a low number of pups in the litters of female rats fertilized by finasteride-treated male rats. Therefore, we had to repeat the procreation process many times, and based on the literature, the reason was probably weak semen quality (as a “*side-effect*” of finasteride). We also observed the elimination of newborns by female rats (infanticide exists when mothers tend to kill deformed, sick, or weak young pups, only allowing the healthy ones to survive [25]). Moreover, in our experiment, female pups predominated in many litters. As was mentioned above, even a low dose of finasteride can exert a negative effect on sperm DNA integrity [15,16,17]; thus, the unequal distribution of sexes in the litters may have been caused by genetic changes in the spermatozoa chromosomes in the finasteride-treated rats. Possibly, finasteride-like endocrine disruptors (EDs), that mimic or block sex hormones, disrupt hormones homeostasis, and can negatively influence reproductive system development and function [26,27], could induce a transgenerational effect. For example, it was shown that pollutants present in semen could cause DNA oxidative damage [28], and molecular alterations in spermatozoa of men living in highly polluted areas are a result of the transgenerational effects of pollutants [29]. The treatment of male mice with dioxin (TCDD, 2, 3, 7, 8-tetrachlorodibenzo-p-dioxin) produced alternations in the sex ratio of the filial generation [30], or the exposure of young men to environmental dioxins increased the probability of female births [31]. Additionally, the changed gender distribution strongly correlated with paternal dioxin exposure rather than maternal exposure [32]. Moreover, Anway et al. [33] showed that in utero exposure of rat fetuses to vinclozolin (an anti-androgenic endocrine disruptor) resulted in a decrease in the number of male pups per litter in subsequent F1–F4 generations, similar to our F1:Fin generation [24].

For the reasons described above, the goal of this work was to check if the testicular (without distinguishing cellular populations) transcriptome profile in the male filial generation (F1:Fin) from paternal rats (F0:Fin) receiving finasteride had changed.

## 2. Material and Methods

### 2.1. Animals

The study was conducted with albino Wistar rats in full accordance with Polish law and with the approval of the Local Ethics Committee for Scientific Experiments on Animals in Szczecin, Poland (Resolution No. 23/2010 dated 21 July 2010). Maximum efforts were made to minimize the number of animals and their suffering. The parental generation (F0) and their offspring (F1) were termed.

#### 2.1.1. The Parental Generation

The parental generation was comprised of 12 female and 10 male rats (for mating purposes only). At the beginning of the experiment, the rats were 12 weeks old. During the 1 week adaptation period, the animals were housed in cages (3 females or 1 male per cage) in the Animal Facility of the Pomeranian Medical University. The room humidity was approximately 55%, and the air temperature 22 ± 2 °C. The lighting followed a 12/12 h day/night cycle. After the adaptation, the males were randomly divided into two groups (F0:Control, *n* = 4; and F0:Fin, *n* = 6). The six male rats of the F0:Fin group were administered finasteride (Proscar^®^, MSD, Carmlington, UK) at 5 mg/kg/bw once a day (in the morning) as a small pellet of finasteride powder placed in bread. These animals willingly ate the pellets from the hand of the person performing the experiments. The paternal control group (F0:Control) received the same bread pellet but without finasteride. The dose of finasteride was the same as in an earlier investigation [34] and as described by others [35,36]. The period of finasteride treatment before mating lasted 56 days, in line with the results of our previous study, which had demonstrated pathomorphological changes in the seminiferous epithelium [34], and continued up to the end of the experiment (for 4–5 months). Once a week, the animals were weighed, and the finasteride dose was suitably adjusted. The mating of couples from the F0:Control and F0:Fin groups began at the same time to maintain the age of the parental animals for comparisons. Each mating pair was kept in one cage. After 1 week, the females were separated from the males, and each pregnant female rat was placed in a separate cage. Standard food and tap water were freely available.

#### 2.1.2. The Filial Generation

The control offspring group (F1:Control) comprised male rats born from females fertilized by control male rats. The experimental offspring group (F1:Fin) comprised male rats born from females fertilized by the finasteride-treated male rats. The objective of the experiment was to take samples of the gonads from both F1 generations of rats at 14 and 90 postnatal days (PND). The 14-day-old offspring comprised 3 male rats from each group (F1:Control 14, F1:Fin 14); the 90-day-old offspring comprised the remaining 3 male rats from each of the groups (F1:Control 90, F1:Fin 90). This experimental model of gonad maturity is based on the fact that in the seminiferous epithelium of 7-day-old rats, there are only gonocytes and Sertoli cells, and during maturation (i.e., successive weeks of the experiment), other generations of germ cells occur within the seminiferous epithelium [24,37], which is why we selected gonads from 14-day-old rats. A completely mature gonad with spermatozoa production ability can be found in 3-month-old rats (90 days old) [24]. Following thiopental anesthesia (120 mg/kg bw, i.p., Biochemie GmbH, Wien, Austria), the testes from the offspring in each F1 group were resected and stored at −70 °C for further analysis.

### 2.2. Microarrays Analysis

#### 2.2.1. RNA Isolation

Total RNA from each testis’ homogenates was isolated using a PARIS Kit (Thermo Fisher Scientific, Waltham, MA, USA) according to the manufacturer’s protocol, in four samples for subsequent microarray analysis: control rats at 14 days old (F1:Control 14), control rats at age 90 days old (F1:Control 90), finasteride-treated male parent rat offspring at age 14 days old (F1:Fin 14), finasteride-treated male parent rat offspring at age 90 days old (F1:Fin 90). Total RNA in a homogenate concentration of 100 ng/µL was used for the microarray experiment.

#### 2.2.2. Affymetrix GeneChip Microarray and Data Analysis

A sense-strand cDNA generated from the total RNA was subjected to fragmentation and labeling using the GeneChip™ WT PLUS Reagent Kit (Thermo Fisher Scientific, Waltham, MA, USA) and then hybridized onto a Rat Gene 2.1 ST Array Strip. Hybridization, subsequent fluidics, and scanning steps were performed using an Affymetrix GeneAtlas™ System. Preliminary analysis and quality control of the scanned chips was performed using Affymetrix GeneAtlas Operating Software. The generated CEL files were subjected to further analysis using the R statistical language with the Bioconductor package and relevant libraries. For background correction, normalization, and summation of the raw data, the Robust Multiarray Averaging (RMA) algorithm implemented in the “affy” package of BioConductor was applied. Biological annotation was obtained from the “oligo” package of BioConductor in which the annotated data frame object was merged with the normalized data set, leading to a complete gene data table. The selection criteria for a significantly changed gene expression were based on an expression fold difference greater than |1.5|. Gene expression datasets fulfilling the cut-off criteria were visualized with scatter plots.

The DAVID Direct Database for Annotation, Visualization and Integrated Discovery was used to cluster the functional annotation of differentially expressed genes [38]. Gene symbols for up-regulated and down-regulated genes from each of the compared groups were loaded into DAVID using the “RDAVIDWebService” package of BioConductor. Functional annotation charts generated by DAVID with over-represented gene annotations are shown as bubble plots generated by the BACA package of BioConductor with the following criteria: *p* < 0.05, adjusted method = Benjamini, and minimal number of genes per group = 5. Groups of genes fulfilling these criteria are presented in a plot in which the bubble sizes indicate the number of genes represented in the corresponding annotation and the condition of these genes in terms of their downregulation and upregulation. 

## 3. Results

### 3.1. Differential Gene Expression Profiles

**Figure 1:**
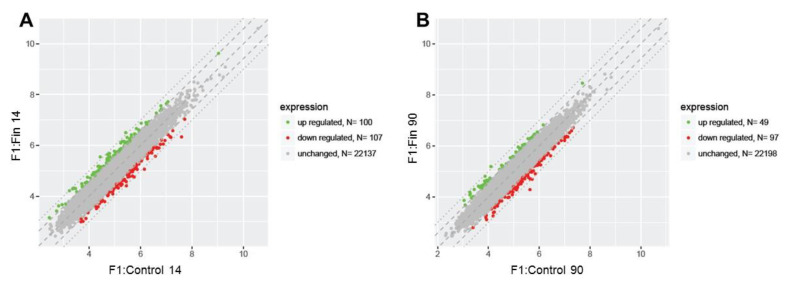
Scatter plots illustrating differentially expressed genes (DEGs) in the four experimental comparison groups: F1:Fin 14 vs. F1:Control 14 (**A**), F1:Fin 90 vs. F1:Control 90 (**B**). Each dot represents a single gene. The selection criteria for significantly changed gene expression were based on a greater than 1.5-fold difference in expression.

**Figure 2:**
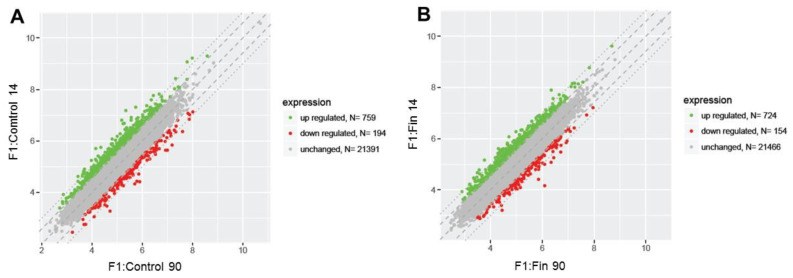
Scatter plots illustrating differentially expressed genes (DEGs) in the four experimental comparison groups: F1:Control 14 vs. F1:Control 90 (**A**), F1:Fin 14 vs. F1:Control 90 (**B**). Each dot represents a single gene. The selection criteria for significantly changed gene expression were based on a greater than 1.5-fold difference in expression.

Figure 1 and Figure 2. show a detailed analysis of the differentially expressed genes (DEGs; differentially expressed genes – fold change ≤ −1.5 (down-regulated genes) and fold change ≥ 1.5 (up-regulated genes)) in the testis from the control and experimental groups of rats at 14 days old (F1:Control 14, F1:Fin F14) and 90 days old (F1:Control 90, F1:Fin 90). The scatter plots in Figure 1A,B and Figure 2A,B show the four comparisons groups (F1:Fin 14 vs. F1:Control 14, F1:Fin 90 vs. F1:Control 90, F1:Control 14 vs. F1:Control 90, F1:Fin 14 vs. F1:Fin 90).

We found that a total of 22344 genes (in each comparison) were regulated in the immature rats F1:Fin 14 vs. F1:Control 14: 100 up-regulated, 107 down-regulated, and 22137 unchanged (Figure 1A). In the sexually mature individuals (F1:Fin 90 vs. F1:Control 90), 49 genes were up-regulated, 97 down-regulated, and 22198 unchanged (Figure 1B).

During physiological aging (Figure 2A,B), both in control (F1:Control) and experimental (F1:Fin) groups, a very similar number of genes were up-regulated (759 and 724, respectively), down-regulated (194 and 154, respectively) or unchanged (21391 and 21466, respectively).

### 3.2. List of the Top 10 Changed Genes

The top 10 genes with the highest and lowest fold changes from each of the four comparisons (F1:Fin 14 vs. F1:Control 14, F1:Fin 90 vs. F1:Control 90, F1:Control 14 vs. F1:Control 90, F1:Fin 14 vs. F1:Fin 90) are listed in Table 1. It should be noted that the analyzed transcriptome came from testicular tissue that contains different, heterogenic populations of cells (e.g., Sertoli, Leydig, germ cell, etc.).

Among the four comparisons mentioned above, and from the listed 80 top genes, 17 genes encode odoriferous receptors (*Olfr*: *31*, *331*, *365* x2, *633*, *774*, *814*, *890*, *935*, *1109*, *1112* x2, *1173*, *1251*, *1259*, *1253*, *1383*), 8 genes encode vomeronasal receptors (*Vmn1r*: *50*, *103*, *210*, *211* x2; *Vmn2r*: *3*, *23*, *99*) and 1 gene encodes *RIKEN cDNA 5430402E10*, which is also known as odorant-binding protein 2. These are the most extensively altered group of genes concerning G-protein-coupled receptors acting in the sensory perception of smell (odorant substances and pheromones).

Comparing the 14-day-olds (F1:Control 14 vs. F1:Fin 14), among the odoriferous receptors genes, five were up-regulated (*Olfr365*, *Olfr814*, *Olfr774*, *Olfr935*, *Olfr31*) and two down-regulated (*Olfr633*, *Olfr1112*), while among the vomeronasal receptors, one gene was up-regulated (*Vmn1r211*) and one down-regulated (*Vmn2r23*). Comparing the two groups of animals at 90 days old (F1:Control 90 vs. F1:Fin 90), among the odoriferous receptor genes, three genes were up-regulated (*Olfr1109*, *Olfr1212*, *Olfr1173*) and one was down-regulated (*Olfr1383*); three genes of vomeronasal receptor were up-regulated.

Additionally, microarray analysis shows age-related changes (14 days old vs. 90 days old) in transcript expression. In the control animals, two genes were up-regulated (*Olfr1259*, *Olfr331*) and three down-regulated (*Olfr365*, *Vmn1r211*, *Vmn2r99*). In the F1:Fin group of rats, three genes were up-regulated (*Olfr1253*, *Vmn2r3*, *RIKEN cDNA 5430402E10*) and one was down-regulated (*Olfr890*, *Olfr1251*).

In the comparisons—F1:Control 14 vs. F1:Fin 14 and F1:Control 90 vs. F1:Fin 90—among the genes directly related to fertility, changes were found in: *Glipr1l1* (up-regulated; plays a role inter alia in the binding between sperm and oocytes), *Rln1* (down-regulated; important, e.g., for prostate gland growth and spermatogenesis); and for genes indirectly related to fertility, changes were found in: *Ugt2b5* (up-regulated; e.g., catalyzes the glucuronidation of endogenous steroid hormones, cellular response to testosterone stimulus, estrogen metabolic process that occur, e.g., in Sertoli cells), *Akr1c19* (up-regulated; has steroid dehydrogenase activity), *Vmn1r50* (up-regulated; putative pheromone receptor implicated in the regulation of social and reproductive behavior), *Bod1* (down-regulated; need to proper cell division), *Wdr83os* (up-regulated; post-embryonic development), *Ormdl3* (down-regulated; positive regulation of autophagy, e.g., elimination of residual bodies by Sertoli cells), *Dsg1c* (down-regulated; cell–cell adhesion, e.g., germ cells to Sertoli cells), *Spin2d* (down-regulated; regulation of cell cycle, gamete generation), *Gm5132* (down-regulated; chromatin silencing), *Tmem161a* (up-regulated; negative regulation of intrinsic apoptotic signaling pathway in response to DNA damage, positive regulation of DNA repair).

Age-related (14- vs. 19-day-old) changes in gene expression (with the exception of receptors related to the perception of odors) in the F1:Control group were shown mainly in genes associated with immune defense: *C1rb* (up-regulated; complement activation, innate immune response), *Trbj1-6* (down-regulated; essential for T cell growth and differentiation), regulation of transcription: *Ssxb2* (down-regulated; acts as a modulator of transcription), and DNA repair: *Bod1* (up-regulated; DNA repair), *Rex2* (down-regulated; DNA repair, replication, recombination), and *Idi2* (up-regulated, cholesterol biosynthesis) needed for steroidogenesis. Age-related changes in gene expression in the F1:Fin group showed some similarities in the genes associated with immune defense (but different genes than in the F1:Control rats): *Igkv1-110* (up-regulated; immune response), *Gsdmc2* (up-regulated; defense response to bacteria); and fertility: *Rln1* (down-regulated; prostate gland growth, regulation of cell population proliferation, and spermatogenesis) and *Dsg1c* (down-regulated; cell–cell adhesion, e.g., germ cells to Sertoli cells). Therefore, many changed genes that were related to immune defense could probably be due to the fact that the interstitial tissue of the testes included many lymphatic vessels.

**Figure 3:**
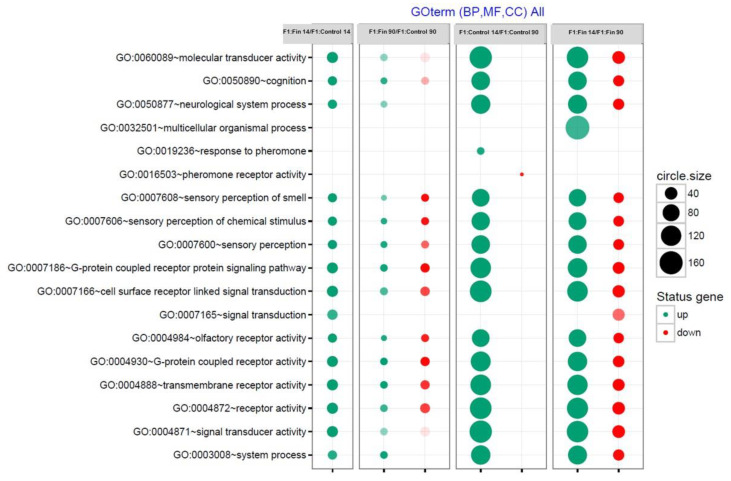
Biological process, molecular function, and cellular component assigned according to the Gene Ontology (GO) classification for DEGs found in homogenates of testicular tissue from four groups of experimental rats; the comparisons were made in F1:Fin or F1:Control group in relation to age (F1:Control 14/F1:Control 90; F1:Fin 14/F1:Fin 90) or between experimental and control group in relation to their age (F1:Fin 14/F1:Control 14; F1:Fin 90/F1:Control 90). Groups of genes fulfilling the criteria: adjusted *p* < 0.05, method = Benjamini, and minimum number of genes per group = 5, are presented in a graph in which the bubble size indicates the number of genes represented in the corresponding annotation and the condition of these genes in terms of their up- and down-regulation. The transparency of the bubbles reflects the *p*-value (more transparent is closer to the border of *p* = 0.05).

Subsequently, up-regulated and down-regulated genes (DEGs) from all experimental comparison groups were assigned a Gene Ontology term for the biological process, molecular function, and cellular component (GO term MF, BP, CC) per Benjamini method, as shown in Figure 3.

The bubble plots demonstrate that the greatest changes in gene expression correlate with age: in the control animals, this expression was mostly up-regulated, while in the finasteride-treated male offspring (F1:Fin), there were both up-regulation and down-regulation. When comparing the 14-day-old groups, F1:Fin 14 to F1:control 14, the expression was up-regulated, while in the 90-day-old groups, F1:Fin 90 vs. F1:Control 90, the expression was both up-regulated and down-regulated, but in a lower degree than in the age-dependent comparison.

## 4. Discussion

The microarray analysis of the testicular transcriptome profile shows that the top 10 genes with the highest and lowest changes in fold expression (F1:Fin 14 vs. F1:Control 14, F1:Fin 90 vs. F1:Control 90, F1:Control 14 vs. F1:Control 90, F1:Fin 14 vs. F1:Fin 90) were mainly genes associated with smelling (olfactory receptors, vomeronasal receptors).

It is known that inter alia sensory processing modulation induces reproductive behavior and endocrine changes. The microarray analysis shows that vomeronasal 1 receptor 50 (*Vmn1r50* a G-protein coupled receptor) in mature male F1:Control 90 rats was up-regulated in comparison to the F1:Fin 90 group. This receptor is a putative pheromone receptor implicated in the regulation of social and reproductive behavior (https://www.uniprot.org/uniprot/Q9EP51; accesse date: 24 June 2021).

Across the animal kingdom, there are evolved sensory and behavioral strategies to identify suitable mating partners to guarantee reproductive success. It is known that odoriferous substances called pheromones carry species-specific and gender-specific information necessary for mating [39]. In this respect, reproductive substances play a very important role and generally have a negative effect on reproductive function after reaching a critical age. In more highly derived vertebrate clades (i.e., amniotes), a special male vomeronasal organ (Jacobson’s organ) evolved for the detection of pheromones [40]. Therefore, the changes observed by us in the transcriptome of F1:Control vs. F1:Fin in the rat offspring, in the context of pheromone receptors, could suggest some dysfunction in the evolutionary sexual behavior.

Furthermore, and more importantly for spermatozoa function, because our research material was testicular tissue, odoriferous substances are not only involved in the reproductive behavior of animals, but it has also been documented that olfactory-like signaling mechanisms are required for proper sperm physiology [41]. In the 1990s, olfactory receptor (OR) expression was uncovered in various ectopic tissues, including the testes and sperm cells [41]. Canine and human male germ cells demonstrate transcripts of about 20 OR genes, mostly during the late stages of spermatogenesis and during epididymal maturation [42], and ORs are placed in the midpiece and base of the flagellum of mature sperm [43]. Zhang et al. [44] used microarrays to detect 66 OR genes in mouse testes. Therefore, a hypothesis was put forward that the OR expression in male germ linage cells suggesting their potential implication in the control of sperm maturation, migration, and the chemical communication for sperm/egg fertilization [45], which is finding more and more supporting evidence [41,46,47].

One of the evidences that the reproductive potential of the animals treated with finasteride can be impaired is the publication by Kolasa-Wołosiuk et al. [24]. It was observed that the number of pups was lower in the litters of rats fertilized by finasteride-treated male rats, and repetition of the procreation process was needed. In this group of animals, infanticides and a gender shift in favor of females could have been the result of poor quality of sperm in the paternal generation. The results of the microarrays analysis of the testis of the male offspring evidenced a changed pattern in the expression of various olfactory receptors. This could indicate some physiological problems in the spermatozoa of those animals because inter alia olfactory receptors have been shown to mediate spermatozoa chemotaxis [47]. *OR1D2* (Olfactory Receptor Family 1 Subfamily D Member 2, also known as *hOR17-4*) was the first odoriferous receptor expressed in the testes that was demonstrated to be involved in chemotaxis [46]. Ottaviano et al. [47] conducted a study on nine healthy normozoospermic adult males (34.6 ± 3.8 years) who had not been able to have children in the last 3 years. In the control group of men who already had children, this study showed a significantly higher percentage of spermatozoa had migrated toward capillaries filled with bourgeonal (the most potent known agonist of OR1D2) compared to the nine healthy childless men. Thirteen single nucleotide polymorphisms of the *OR1D2* gene in the men suffering unexplained infertility were documented. Therefore, theoretically, idiopathic infertility could be caused by the improper action of odoriferous receptors [47].

Spermatozoa traveling to the oocyte are guided along the female internal reproductive tract by thermotaxis, chemotaxis, and molecules enhancing chemoattraction [48]. The sources of biochemically active biomolecules that facilitate the spermatozoa forward motility, sperm selection/survival, and oocyte–sperm communication are the ovaries (the oocyte and cells of *cumlus oophorus* secrete into the follicular fluid sperm chemoattractants) [49] and the female reproductive tract [41,46,50]. Moreover, the ovarian follicles could also communicate with the spermatozoa prior to ovulation [51]. Therefore, follicular fluid as well as oviduct fluid not only provide a proper microenvironment and nutritional status for the development of oocytes and then their transport, but also afford sperm hyperactivation (needed for proper movement), their navigation via the mucus-filled narrow labyrinthine lumen of the fallopian tube, and finally, penetration of the zona pellucida to cross-talk with the oocyte [52]. It was documented that the movement pattern of spermatozoa has been controlled differently by the composition of metabolites in the fluid milieu present in the female reproductive tract [53]. Additionally, some critical regulators secreted from the ovary and reproductive tract play a crucial role in determining oocyte–sperm interaction and communication in a dose-dependent manner [54]. Undoubtedly, many studies have presumed the involvement of odorant-like signaling in the initiation of spermatozoa hyperactivation; however, only a few have been identified to date [53]. In the light of the above-described reports, the changes that have been demonstrated in this present study in the transcriptome of F1:Fin rats, in the context of olfactory receptors and vomeronasal receptors, may assume impaired spermatozoa physiology (chemotaxis, swimming speed, hyperactive flagella beating, cross-talk with oocyte). Our previous study showed changes in the severity of apoptosis in the seminiferous epithelium of rats from F1:Fin vs. F1:Control [24], possibly related to the fact that some olfactory receptors are also involved in specific functions underlying sperm maturation and transport them between Sertoli cells from the basal membrane to the seminiferous tubular lumen, in which reduced expression has linked them to male infertility in azoospermic patients with spermatocyte development arrest [55,56].

The Benjamini method of transcriptome analysis shows that in the F1:Fin male offspring, the molecular function and the cellular component were down-regulated with time, which is likely why we previously had problems with the fertilization of female rats when using finasteride-treated male rats [24]. Certainly, the question “*Can spermatozoa smell?”* is still open, and new discoveries will probably unravel the mysteries of odoriferous and vomeronasal receptor expression in mammalian sperm. The nature and source of such potential chemical guideposts, as well as the molecular mechanisms underlying chemically induced changes in sperm motility, are still unknown.

The literature data on the effect of finasteride on the expression of androgen receptors (AR) are contradictory [57,58]. However, the discrepancies can be explained by the different types of tested tissues and by biochemical factors. Additionally, the polymorphisms of the AR genes have been described [59], among which those that increase the probability of the occurrence of the post-finasteride syndrome or androgenic alopecia have been identified [60]. Our previous work [61] showed that in the liver obtained from the offspring derived from treated with finasteride paternal generation, expression of AR on mRNA level was statistically reduced only in adult F1:Fin rats.

Our work focuses on the description of the observed changes. However, based on the literature, we can describe the possible mechanisms that lead to their formation. Especially that the finasteride-induced epigenetic changes in gene expression, including changes of androgen receptors expression, increased histone acetylation and methylation, result in undesirable biological effects [62]. Some papers [63] indicate that the likely mechanism of transgenerational action of finasteride can be connected with CpG methylation in the 5α-reductase type 2 gene and consequently change its protein expression in prostatic tissue. Traish [62], based on a literature review, proposed the following steps of finasteride induced epigenetic changes: (i) finasteride binds to the 5α-red2 with high affinity; (ii) at the same time, finasteride dissociates very slowly from 5α-red2; (iii) turnover of the 5α-red2 is short; (iv) in this situation, it may take up to 4–8 weeks for the physiological level of 5α-DHT to recover; (v) the key endocrine pathway (including hypothalamic-pituitary axis) is disrupted and prolonged 5α-DHT deficiency can induce changes; (vi) silencing of 5α-red2 expression and altering expression and androgen function is likely; (vii) additionally, finasteride leads to an inhibition of neurosteroid biosynthesis, which may exacerbate epigenetic changes. Thus, epigenetic modifications induced by finasteride may result in suppression not only of the hypothalamic–pituitary–testicular axis, irreversible suppression of 5α-reductase expression, off-target suppression of androgen receptor action, but also effects on brain regions that regulate sexual function and mood [62].

There are no studies on the direct effect of finasteride/DHT deficiency on the expression of odorant receptors. However, it was documented that disruption of a critical endocrine pathway may lead to induced epigenetic changes in response to 5α-DHT deficiency, which may contribute to the silencing of the 5α-red2 gene expression and changes in AR expression and function [62]. In seminiferous epithelium, both Sertoli, as well as germ cells, express androgen receptors [64,65], so probably, the changed AR expression in the testes of F1:Fin rats (as we observed this change in the liver of these rats [61]) could result in improper physiology of seminiferous epithelium or spermatozoa.

As with any scientific publication, our work also has limitations. First of all, the transcriptome was evaluated in the testicular tissue without distinguishing cellular populations (Leydig, Sertoli, germ cells, etc.) Our primary focus is on the changes observed in the offspring of paternal rats administered finasteride. We have no evidence of what mechanisms led to these changes. Therefore, we can only make assumptions and plan research on these mechanisms in the future. We do not have marked the level of protamination of sperm DNA, an important marker of stabilization of chromatin structure and maturity of sperm; we will try to perform such analyses in the future.

Among other genes associated with male fertility that were up-regulated or down-regulated between the F1:Fin vs. F1:Control rats, we found the genes that are involved in: (i) binding between sperm and oocytes; (ii) prostate gland growth and spermatogenesis controlled by Sertoli cells; (iii) cellular response to testosterone stimulus and estrogen metabolic process (that occur, e.g., in Sertoli cells); (iv) proper cell division/cell cycle (gamete generation), chromatin silencing, regulation of intrinsic apoptotic signaling pathway in response to DNA damage; (v) regulation of autophagy, e.g., during the elimination of residual bodies by Sertoli cells; and (vi) cell–cell adhesion, e.g., germ cells to Sertoli cells. Thus, it can be hypothesized that the rat offspring in the F1:Fin group may be characterized by impaired spermatogenesis controlled and regulated by Sertoli cells in response to steroid hormones, spermatozoa physiology, or some under-development in the male reproductive system or other organs. Additionally, during aging, the transcriptome profile for genes encoding proteins involved in the immune response was also changed, but these genes were different in the F1:Control group than in the F1:Fin group.

## 5. Conclusions

Finasteride treatment of male adult rats may cause changes in the testicular transcriptome profile of their offspring, leading to a defective function of germinal and somatic cells (Leydig, Sertoli cells, etc.) in response to odorant-like signals, which are recently more and more often noticed as significant players in male fertility.

## Figures and Tables

**Table 1:** Top 10 genes with the highest and lowest values of the fold expression change within the four individual comparison groups: F1:Control 14 vs. F1:Fin 14, F1:Control 90 vs. F1:Fin 90, F1:Control 14 vs. F1:Control 90, F1:Fin 14 vs. F1:Fin 90. Characteristic of gene/pseudogene function mainly from uniprot.org (accesse date: 24 June 2021) or ncbi.nlm.nih.gov (accesse date: 24 June 2021) and genecards.org (accesse date: 24 June 2021).

Comparison Group	Gene Symbol	Gene/Pseudogene Name and Its Major Characteristic/Function	Entrez Gene ID	Fold Change
F1:Fin 14 vs. F1:Control 14	*Olfr365*	*olfactory receptor 365*; olfactory receptor, odorant binding. G-protein-coupled receptor activity, olfactory receptor activity. Sensory perception of smell (https://www.uniprot.org/uniprot/Q8VFT2; accesse date: 24 June 2021).	405932	2.16
	*Olfr814*	*olfactory receptor 814*; olfactory receptor, odorant binding. G-protein-coupled receptor activity, olfactory receptor activity. Sensory perception of smell (https://www.uniprot.org/uniprot/Q7TRH4; accesse date: 24 June 2021).	405135	2.11
	*Ugt2b5*	*UPD glucuronosyltransferase 2 family, (UGT)*; increase lipophilic substrates water solubility. Glucuronidation of steroid hormones. Cellular response to glucocorticoid, growth hormone, testosterone stimulus; estrogen metabolic process (https://www.uniprot.org/uniprot/P08542; accesse date: 24 June 2021).	266685	1.98
	*Olfr774*	*olfactory receptor 774*; olfactory receptor, odorant binding. G-protein-coupled receptor activity, olfactory receptor activity. Sensory perception of smell (https://www.uniprot.org/uniprot/Q7TRI5; accesse date: 24 June 2021).	405233	1.91
	*Vmn1r211*	*vomeronasal 1 receptor 211*; G-protein-coupled receptor. Pheromone receptor activity, pheromone binding, response to pheromone (https://www.uniprot.org/uniprot/Q8R266; accesse date: 24 June 2021).	494278	1.90
	*Olfr935*	*olfactory receptor 935*; olfactory receptor, odorant binding. G-protein-coupled receptor activity, olfactory receptor activity. Sensory perception of smell (https://www.uniprot.org/uniprot/Q8VG16; accesse date: 24 June 2021).	288868	1.89
	*Olfr31*	*olfactory receptor 31*; olfactory receptor, odorant binding. G-protein-coupled receptor activity, olfactory receptor activity. Sensory perception of smell (https://www.uniprot.org/uniprot/E9Q3K2; accesse date: 24 June 2021).	405403	1.85
	*Krtap19-9b*	*keratin associated protein 19-9B*; hair keratin filaments are embedded in matrix, consisting of hair keratin-associated proteins (KRTAP), which are essential for the formation of a rigid and resistant hair shaft (https://www.uniprot.org/uniprot/Q99NG9; accesse date: 24 June 2021).	360626	1.83
	*Zfp874a*	*zinc finger protein 874a* or *regulator of sex-limitation candidate 15*; DNA-binding transcription factor activity. Regulation of transcription by RNA polymerase II (https://www.uniprot.org/uniprot/Q8BX23; accesse date: 24 June 2021).	286979	1.82
	*Glipr1l1*	*GLI pathogenesis-related 1 like 1*; binding of sperm to zona pellucida; a role in the binding between sperm and oocytes. Part of epididymosomes, membranous microvesicles which mediate the transfer of lipids and proteins to spermatozoa plasma membrane during epididymal maturation. Component of the CD9-positive microvesicles, found in the cauda region (https://www.uniprot.org/uniprot/Q9DAG6; accesse date: 24 June 2021).	299783	1.80
	*Vmn2r23*	*vomeronasal 2*, *receptor 23*; G-protein-coupled receptor activity (https://www.uniprot.org/uniprot/E9PXI5; accesse date: 24 June 2021).	308303	−1.78
	*Rln1*	*relaxin 1*; acts with estrogen to produce dilatation of the birth canal in many mammals. Hormone activity, signaling receptor binding. Modulation of G-protein-coupled receptor signaling pathway; developmental growth, mammary gland morphogenesis, negative regulation of apoptotic process, involved in nipple development, prostate gland growth, regulation of body fluid levels, regulation of cell population proliferation, regulation of nitric oxide-mediated signal transduction, spermatogenesis (https://www.uniprot.org/uniprot/P01347; accesse date: 24 June 2021).	25616	−1.78
	*Bod1*	*biorientation of chromosomes in cell division 1*; protein phosphatase inhibitor activity. Cell division, mitotic metaphase plate congression, mitotic sister chromatid biorientation and cohesion, centromeric, negative regulation of phosphoprotein phosphatase activity, protein localization to chromosome, centromeric region (https://www.uniprot.org/uniprot/Q6AYJ2, accesse date: 24 June 2021).	287173	−1.82
	*Olfr633*	*olfactory receptor 633*; olfactory receptor, odorant binding. G-protein-coupled receptor activity, olfactory receptor activity. Sensory perception of smell (https://www.uniprot.org/uniprot/Q8VF02; accesse date: 24 June 2021).	404844	−1.83
	*Olfr1112*	*olfactory receptor 1112*; olfactory receptor, odorant binding. G-protein-coupled receptor activity, olfactory receptor activity. Sensory perception of smell (https://www.uniprot.org/uniprot/A2ATA0; accesse date: 24 June 2021).	405559	−1.86
	*Krt17*	*keratin 17*—*type I keratin*; formation and maintenance of various skin appendages (by modulating TNF-alpha, Akt/mTOR, immune response in skin). Involved in tissue repair, a marker of epithelial ‘stem cells’. Constituent of cytoskeleton, hair follicle morphogenesis, intermediate filament-based process, keratinization, morphogenesis of epithelium, positive regulation of cell growth, hair follicle development, and translation (https://www.uniprot.org/uniprot/Q6IFU8, accesse date: 24 June 2021).	287702	−1.99
	*Oas1g*	*2-5 oligoadenylate synthetase 1G*; defense response to viruses, innate immune response, negative regulation of viral genome replication, regulation of ribonuclease activity (https://www.uniprot.org/uniprot/Q5MYX1; accesse date: 24 June 2021).	100910735	−2.01
	*Dsg1c*	*desmoglein 1 gamma*; component of intercellular desmosome junctions. Involved in homophilic cell adhesion via plasma membrane adhesion molecules. Calcium ion binding, gamma-catenin binding (https://www.uniprot.org/uniprot/Q7TSF0; accesse date: 24 June 2021).	291755	−2.06
	*Akr1c19*	*aldo-keto reductase family 1, member C19*; enzymatic activity, including steroid dehydrogenase activity. Involved in steroid metabolic process. 90% identity with NAD-preferring dehydrogenase for 17-β-hydroxysteroids and 9-hydroxyprostaglandins (https://www.uniprot.org/uniprot/D3ZEL2; accesse date: 24 June 2021).	307096	−2.13
	*Gm4340*	*predicted gene 4340*; RNA binding (https://www.uniprot.org/uniprot/L7N2C4; accesse date: 24 June 2021).	100043292	−2.40
F1:Fin 90 vs. F1:Control 90	*Vmn1r103*	*vomeronasal 1 receptor 103*; pheromone receptor activity, response to pheromones (https://www.uniprot.org/uniprot/K7N6X7; accesse date: 24 June 2021).	100312735	2.13
	*Olfr1109*	*olfactory receptor 1109*; olfactory receptor, odorant binding. G-protein-coupled receptor activity, olfactory receptor activity. Sensory perception of smell (https://www.uniprot.org/uniprot/A2AT96; accesse date: 24 June 2021).	405557	1.92
	*Olfr1212*	*olfactory receptor 1212*; olfactory receptor, odorant binding. G-protein-coupled receptor activity, olfactory receptor activity. Sensory perception of smell (https://www.uniprot.org/uniprot/Q7TR08; accesse date: 24 June 2021).	405579	1.89
	*Chil3*	*chitinase-like 3*; binds chitin and heparin. Has chemotactic activity. May play a role in inflammation and allergies. Enzymatic activity, chitin catabolic process, inflammatory response, polysaccharide catabolic process (https://www.uniprot.org/uniprot/O35744; accesse date: 24 June 2021).	295351	1.80
	*Olfr1173*	*olfactory receptor 1173*; olfactory receptor, odorant binding. G-protein-coupled receptor activity, olfactory receptor activity. Sensory perception of smell (https://www.uniprot.org/uniprot/Q7TR24; accesse date: 24 June 2021).	404329	1.79
	*Vmn1r210*	*vomeronasal 1 receptor 210*; pheromone receptor activity, response to pheromones (https://www.uniprot.org/uniprot/Q8R274; accesse date: 24 June 2021).	286957	1.77
	*Gm15056*	*predicted gene 15056*; lack of information (https://www.uniprot.org/uniprot/O88242; accesse date: 24 June 2021).	100504014	1.76
	*Tmem161a*	*transmembrane protein 161A*; cellular response to oxidative stress, cellular response to UV, negative regulation of intrinsic apoptotic signaling pathway in response to DNA damage, positive regulation of DNA repair, response to retinoic acid (https://www.uniprot.org/uniprot/B1WC35; accesse date: 24 June 2021).	364535	1.75
	*Wdr83os*	*WD repeat domain 83 opposite strand* (preferred name protein: Asterix); component of the complex that facilitates multi-pass membrane proteins insertion into membranes, including ER. Involved in organization of the ER. Calcium ion homeostasis, ERAD pathway, ER overload response, post-embryonic development (https://www.uniprot.org/uniprot/Q5U2X6; accesse date: 24 June 2021).	288925	1.72
	*Vmn1r50*	*vomeronasal 1 receptor 50*; G-protein coupled receptor, putative pheromone receptor implicated in the regulation of social and reproductive behavior. Pheromone receptor activity, response to pheromone, sensory perception of chemical stimulus (https://www.uniprot.org/uniprot/Q9EP51; accesse date: 24 June 2021).	494267	1.71
	*Hist1h2bn*	*histone cluster 1, H2bn*; transcription regulation, DNA repair, DNA replication, and chromosomal stability. DNA binding, identical protein binding, protein heterodimerization activity. Antibacterial and antimicrobial immune response, defense response to Gram-positive bacterium, innate immune response in mucosa, nucleosome assembly (https://www.uniprot.org/uniprot/P10853; accesse date: 24 June 2021).	291157	−1.77
	*Ptms*	*parathymosin*; mediate immune function by blocking the effect of prothymosin alpha. Enzyme inhibitor activity, zinc ion binding, immune system processes (https://www.uniprot.org/uniprot/P04550; accesse date: 24 June 2021).	83801	−1.77
	*Mrgpra6*	*MAS-related GPR, member A6*—*orphan receptor*; G-protein-coupled receptor activity. A receptor for some neuropeptides, which are analgesic in vivo. Regulate nociceptor function and/or development, the sensation/modulation of pain (https://www.uniprot.org/uniprot/Q91ZC6; accesse date: 24 June 2021).	381886	−1.78
	*Ormdl3*	*ORM1-like 3*; cellular sphingolipid homeostasis, ceramide metabolic process, motor behavior, myelination, negative regulation of B cell apoptotic process, ceramide biosynthetic process, serine C-palmitoyltransferase activity; positive regulation of autophagy, protein localization to nucleus; regulation of ceramide biosynthetic process, smooth muscle contraction, sphingolipid and sphingomyelin biosynthetic process (https://www.uniprot.org/uniprot/Q6QI25; accesse date: 24 June 2021).	360618	−1.79
	*Spin2d*	*spindlin family, member 2D*; methylated histone binding. Regulates cell cycle, transcription, DNA-templated, gamete generation (https://www.uniprot.org/uniprot/B1B0R2; accesse date: 24 June 2021).	100504429	−1.81
	*Olfr1383*	*olfactory receptor 1383*; olfactory receptor, odorant binding. G-protein-coupled receptor, olfactory receptor activity. Sensory perception of smell (https://www.uniprot.org/uniprot/Q7TQT2; accesse date: 24 June 2021).	287232	−1.91
	*Supt4a*	*suppressor of Ty 4A*; regulates mRNA processing and transcription elongation, chromatin organization, mRNA processing, elongation, negative and positive regulation of transcription (https://www.uniprot.org/uniprot/P63271; accesse date: 24 June 2021).	20922	−1.91
	*Gm5132*	*predicted gene 5132*; DNA binding, protein heterodimerization activity. Chromatin silencing (https://www.uniprot.org/uniprot/L7MU04; accesse date: 24 June 2021).	333452	−1.91
	*Rex2*	*RNA Exonuclease 2 Homolog*; 3′-to-5′ exonuclease specific for small single-stranded RNA and DNA oligomers. DNA repair, replication, and recombination, RNA processing, and degradation. Resistance of cells to UV-C-induced cell death. A role in cellular nucleotide recycling (https://www.genecards.org/cgi-bin/carddisp.pl?gene=REXO2; accesse date: 24 June 2021).	104444	−2.04
	*AF067061*	*cDNA sequence AF0670*; lack of information (https://www.uniprot.org/uniprot/O70616; accesse date: 24 June 2021).	236546	−2.55
F1:Control 90 vs. F1:Control 14	*Bod1*	*biorientation of chromosomes in cell division 1*; DNA binding, protein phosphatase 2A binding, protein phosphatase inhibitor activity, cellular response to DNA damage stimulus, DNA repair, negative regulation of phosphoprotein phosphatase activity, replication fork processing (https://www.uniprot.org/uniprot/E9Q6J5; accesse date: 24 June 2021).	287173	3.57
	*AY358078*	*cDNA sequence AY358078* (HN1-like protein); lack of information (https://www.uniprot.org/uniprot/Q6UY53; accesse date: 24 June 2021).	278676	3.09
	*Gm8995*	*predicted gene 8995*; lack of information (https://www.uniprot.org/uniprot/Q8C9P2; accesse date: 24 June 2021).	668139	2.97
	*Klk1b5*	*Kallikrein 1-related peptidase b5*; enzymatic activity, acting on carbon–nitrogen (but not peptide) bonds, proteolysis, regulation of systemic arterial blood pressure, zymogen activation (https://www.uniprot.org/uniprot/P15945; accesse date: 24 June 2021).	16622	2.94
	*Idi2*	*isopentenyl-diphosphate delta isomerase 2*; enzymatic activity, metal ion binding, cholesterol biosynthetic process, isopentenyl diphosphate biosynthetic process, isopentenyl diphosphate metabolic process, negative regulation of cholesterol biosynthetic process (https://www.uniprot.org/uniprot/Q8BFZ6; accesse date: 24 June 2021).	502143	2.70
	*Olfr1259*	*olfactory receptor 1259*; G-protein-coupled receptor activity, olfactory receptor, odorant binding, olfactory receptor activity. Sensory perception of smell (https://www.uniprot.org/uniprot/Q8VEZ1; accesse date: 24 June 2021).	300581	2.64
	*Krt17*	*keratin 17*; described above.	287702	2.64
	*Olfr331*	*olfactory receptor 331*; G-protein-coupled receptor activity, olfactory receptor, odorant binding, olfactory receptor activity. Sensory perception of smell (https://www.uniprot.org/uniprot/Q5NC44; accesse date: 24 June 2021).	405033	2.55
	*Snora5c*	*small nucleolar RNA* (snoRNAs), *H/ACA box 5C*; involved in RNA processing (https://www.ncbi.nlm.nih.gov/gene/677796; accesse date: 24 June 2021).	*	2.50
	*C1rb*	*complement component 1, r subcomponent B*; complement activation, classical pathway, innate immune response, zymogen activation, calcium ion binding, identical protein binding, serine-type endopeptidase activity (https://www.uniprot.org/uniprot/Q8CFG9; accesse date: 24 June 2021).	667277	2.43
	*Trbj1-6*	*T cell receptor beta joining 1-6*; participates in the antigen recognition. Initiates three major signaling pathways, the calcium, the mitogen-activated protein kinase (MAPK) kinase, and the nuclear factor NF-kappa-B (NF-kB) pathways (https://www.genecards.org/cgi-bin/carddisp.pl?gene=TRBJ1-6; accesse date: 24 June 2021).	100125253	−2.18
	*Vmn1r211*	*vomeronasal 1 receptor 211*; described above.	171277	−2.21
	*Vmn2r99*	*vomeronasal 2, receptor 99;* G-protein-coupled receptor activity (https://www.uniprot.org/uniprot/H3BK37; accesse date: 24 June 2021).	680992	−2.36
	*Gm10503*	*predicted gene 10503*; lack of information (https://www.uniprot.org/uniprot/Q3UUQ9; accesse date: 24 June 2021).	100038436	−2.44
	*Rpl35*	*ribosomal protein L35*; component of the large ribosomal subunit. mRNA binding, ribonucleoprotein complex binding, structural constituent of ribosome, cellular response to UV-B, maturation of transcript, translation (https://www.uniprot.org/uniprot/P17078; accesse date: 24 June 2021).	296709	−2.54
	*Olfr365*	*olfactory receptor 365*; G-protein-coupled receptor activity. Olfactory receptor, odorant binding, olfactory receptor activity. Sensory perception of smell (https://www.uniprot.org/uniprot/Q8VFT2; accesse date: 24 June 2021).	405932	−2.55
	*Apol9a*	*apolipoprotein L 9a*; lipid binding and transport, lipoprotein metabolic process (https://www.uniprot.org/uniprot/Q5XIB6; accesse date: 24 June 2021).	503164	−2.57
	*Ssxb2*	*synovial sarcoma*, *X member B, breakpoint 2*. A modulator of transcription, DNA-templated (https://www.uniprot.org/uniprot/Q8C5Z3; accesse date: 24 June 2021).	387132	−2.58
	*Gm5592*	*predicted gene 5592*; lack of information (https://www.uniprot.org/uniprot/Q3V0A6; accesse date: 24 June 2021).	434172	−2.72
	*LOC102638448*	*40S ribosomal protein S7-like*; lack of information (https://www.uniprot.org/uniprot/?query=LOC102638448&sort=score; accesse date: 24 June 2021).	102638448	−2.80
F1:Fin 90 vs. F1:Fin 14	*Snora5c*	*small nucleolar RNA*, *H/ACA box 5C*; Described above (https://www.ncbi.nlm.nih.gov/gene/677796; accesse date: 24 June 2021).	*	2.93
	*5430402E10Rik*	*RIKEN cDNA 5430402E10* (odorant-binding protein 2); odorant binding, small molecule binding (https://www.uniprot.org/uniprot/Q9D3N5; accesse date: 24 June 2021).	71351	2.63
	*Apol7b*	*apolipoprotein L 7b*; lipid binding and transport, lipoprotein metabolic process (https://www.uniprot.org/uniprot/B1AQP7; accesse date: 24 June 2021).	278679	2.56
	*Vmn2r3*	*vomeronasal 2, receptor 3*; G-protein-coupled receptor activity, olfactory receptor activity (https://www.uniprot.org/uniprot/H3BJ88; accesse date: 24 June 2021).	502213	2.47
	*Igkv1-110*	*immunoglobulin kappa variable 1-110*; immune response (https://www.uniprot.org/uniprot/A0A0B4J1I0; accesse date: 24 June 2021).	381777	2.44
	*Olfr1253*	*olfactory receptor 1253*; G-protein-coupled receptor activity. Olfactory receptor, odorant binding, olfactory receptor activity. Sensory perception of smell (https://www.uniprot.org/uniprot/A2AUA2; accesse date: 24 June 2021).	405091	2.32
	*9030025P20Rik*	*RIKEN cDNA 9030025P20 gene*; lack of information (https://www.uniprot.org/uniprot/Q3TV04; accesse date: 24 June 2021).	100041574	2.27
	*Gsdmc2*	*gasdermin C2*; constitutes the precursor of the pore-forming protein that causes membrane permeabilization and pyroptosis, binds to membrane lipids. Defense response to bacterium, intestinal epithelial cell development (https://www.uniprot.org/uniprot/Q2KHK6; accesse date: 24 June 2021).	331063	2.26
	*Cpq*	*carboxypeptidase Q*; enzymatic activity, liberation of thyroxine hormone, metal ion binding, peptide catabolic process, proteolysis, tissue regeneration (https://www.uniprot.org/uniprot/Q6IRK9; accesse date: 24 June 2021)	58952	2.25
	*LOC102642621*	*uncharacterized LOC10264262*; lack of information (https://www.uniprot.org/uniprot/?query=LOC102642621&sort=score; accesse date: 24 June 2021).	*	2.23
	*Cyp2d9*	*cytochrome P450, family 2, subfamily d, polypeptide 9*; enzymatic activity. Oxidation, heme, iron ion binding, steroid hydroxylase activity, arachidonic acid metabolic process, exogenous drug catabolic process, organic acid metabolic process, xenobiotic metabolic process (https://www.uniprot.org/uniprot/P11714; accesse date: 24 June 2021).	266684	−2.08
	*Rln1*	*relaxin 1*; described above (https://www.uniprot.org/uniprot/P01347; accesse date: 24 June 2021).	25616	−2.10
	*Gm10503*	*predicted gene 10503*; described above (https://www.uniprot.org/uniprot/Q3UUQ9; accesse date: 24 June 2021).	100038436	−2.11
	*Olfr890*	*olfactory receptor 890;* G-protein-coupled receptor activity. Olfactory receptor, odorant binding, olfactory receptor activity. Sensory perception of smell (https://www.uniprot.org/uniprot/Q7TRD9; accesse date: 24 June 2021).	405511	−2.14
	*Gm9805*	*predicted gene 9805*; lack of information (https://www.uniprot.org/uniprot/O88242; accesse date: 24 June 2021).	100534296	−2.28
	*Olfr1251*	*olfactory receptor 1251*; G-protein-coupled receptor activity. Olfactory receptor, odorant binding, olfactory receptor activity. Sensory perception of smell (https://www.uniprot.org/uniprot/Q7TQZ3; accesse date: 24 June 2021).	300576	−2.36
	*Gm5592*	*predicted gene 5592*; described above (https://www.uniprot.org/uniprot/Q3V0A6; accesse date: 24 June 2021).	434172	−2.38
	*Zfp455*	*zinc finger protein 455*; regulation of transcription (https://www.uniprot.org/uniprot/G3V9J4; accesse date: 24 June 2021).	286979	−2.41
	*Dsg1c*	*desmoglein 1 gamma.* described above (https://www.uniprot.org/uniprot/Q7TSF0; accesse date: 24 June 2021).	291755	−2.73
	*LOC102638448*	*40S ribosomal protein S7-like*; described above (https://www.uniprot.org/uniprot/?query=LOC102638448&sort=score; accesse date: 24 June 2021).	102638448	−3.82

* no number in Entrez Gene ID database on 28 June 2021.

## References

[1] Patrão M.T.C.C., Silva E., Avellar M.C.W. (2009). Androgens and the male reproductive tract: An overview of classical roles and current perspectives. Arq. Bras. Endocrinol. Metabol..

[2] Miller W.L., Auchus R.J. (2011). The Molecular Biology, Biochemistry, and Physiology of Human Steroidogenesis and Its Disorders. Endocr. Rev..

[3] Pratis K., O’Donnell L., Ooi G.T., McLachlan R.I., Robertson D.M. (2000). Enzyme assay for 5α-reductase Type 2 activity in the presence of 5α-reductase Type 1 activity in rat testis. J. Steroid Biochem. Mol. Biol..

[4] Metcalf B.W., Levy M.A., Holt D.A. (1989). Inhibitor of steroid 5-reductase in benign prostatic hyperplasia, male pat-tern baldness and acne. Trends Pharm. Sci..

[5] Chiba K., Yamaguchi K., Li F., Ando M., Fujisawa M. (2011). Finasteride-associated male infertility. Fertil. Steril..

[6] Kaufman K.D. (2002). The Finasteride Male Pattern Hair Loss Group Study. Long term (5-year) multinational experience with finas-teride 1 mg in the treatment of men with androgenic alopecia. Eur. J. Dermatol..

[7] Gupta A.K., Charrette A. (2013). The efficacy and safety of 5α-reductase inhibitors in androgenetic alopecia: A network meta-analysis and benefit–risk assessment of finasteride and dutasteride. J. Dermatol. Treat..

[8] Sato A., Takeda A. (2012). Evaluation of efficacy and safety of finasteride 1 mg in 3177 Japanese men with androge-netic alopecia. J Dermatol..

[9] Gur S., Kadowitz P.J., Hellstrom W.J. (2012). Effects of 5-alpha reductase inhibitors on erectile function, sexual desire and ejaculation. Expert Opin. Drug Saf..

[10] Ganzer C.A., Jacobs A.R., Iqbal F. (2014). Persistent Sexual, Emotional, and Cognitive Impairment Post-Finasteride. Am. J. Men’s Health.

[11] Amory J.K., Wang C., Swerdloff R.S., Anawalt B.D., Matsumoto A.M., Bremner W.J., Walker S.E., Haber-er L.J., Clark R.V. (2007). The effect of 5a-re¬ductase inhibition with dutasteride and finasteride on semen parameters and serum hormones in healthy men. J. Clin. Endocrinol. Metab..

[12] Liu K., Binsaleh S., Lo K., Järvi K. (2008). Propecia-induced spermatogenic failure: A report of two cases. Fertil. Steril..

[13] Irwig M.S., Kolukula S. (2011). Persistent Sexual Side Effects of Finasteride for Male Pattern Hair Loss. J. Sex. Med..

[14] Irwig M.S. (2012). Depressive symptoms and suicidal thoughts among former users of finasteride with persistent sexual side effects. J. Clin. Psychiatry.

[15] Tu H.Y.V., Zini A. (2011). Finasteride-induced secondary infertility associated with sperm DNA damage. Fertil. Steril..

[16] Şalvarci A., Istanbulluoglu O. (2012). Secondary infertility due to use of low-dose finasteride. Int. Urol. Nephrol..

[17] Collodel G., Scapigliati G., Moretti E. (2007). Spermatozoa and Chronic Treatment with Finasteride: A TEM and FISH Study. Arch. Androl..

[18] Post-Finasteride Syndrome Foundation (2015). Global Public Health Advisory–US National Institutes of Health Recognises Post-Finasteride Syndrome. us5.campaign-archive2.com/?u=644fb8b633594fee188a85091&id=9cea0753a4&e=5459eb9419.

[19] National Institutes of Health Genetic and Rare Diseases Information Center (2015). Adverse Events of 5-Alpha-Reductase Inhibitors. http://rarediseases.info.nih.gov/gard/12407/post-finasteride-syndrome/resources/1.

[20] Prahalada S., Tarantal A.F., Harris G.S., Ellsworth K.P., Clarke A.P., Skiles G.L., MacKenzie K.I., Kruk L.F., Ablin D.S., Cukierski M.A. (1997). Effects of finasteride, a type 2 5-alpha reductase inhibitor, on fetal development in the rhesus monkey (Macaca mulatta). Teratology.

[21] Bowman C.J., Barlow N.J., Turner K.J., Wallace D.G., Foster P.M.D. (2003). Effects of in Utero Exposure to Finasteride on Androgen-Dependent Reproductive Development in the Male Rat. Toxicol. Sci..

[22] Ricci G., Martinelli M., Luppi S., Bello L.L., De Santis M., Skerk K., Zito G. (2012). Finasteride and fertility: Case report and review of the literature. J. Drugs Dermatol. JDD.

[23] Ahn K.H., Shin J., Hong S.C., Han J.Y., Lee E.H., Lee J.S., Oh M.J., Kim H.J. (2015). Pregnancy Outcomes with Paternal Exposure to Finasteride, a Synthetic 5-Alpha-Reductase Inhibitor: A Case Series. J. Clin. Toxicol..

[24] Kolasa-Wolosiuk A., Misiakiewicz-Has K., Baranowska-Bosiacka I., Gutowska I., Wiszniewska B. (2015). Androgen levels and apoptosis in the testis during postnatal development of finasteride-treated male rat offspring. Folia Histochem. Cytobiol..

[25] Ebensperger L.A. (2007). Strategies and counterstrategies to infanticide in mammals. Biol. Rev..

[26] Phillips K.P., Foster W.G. (2008). Key Developments in Endocrine Disrupter Research and Human Health. J. Toxicol. Environ. Health Part B.

[27] Cowin P.A., Foster P.M.D., Risbridger G.P., Gore A.C. (2007). Endocrine disruption in the male. Endocrine Disrupting Chemicals. From Basic Research to Clinical Pratice.

[28] Lettieri G., D’Agostino G., Mele E., Cardito C., Esposito R., Cimmino A., Giarra A., Trifuoggi M., Raimondo S., Notari T. (2020). Discovery of the Involvement in DNA Oxidative Damage of Human Sperm Nuclear Basic Proteins of Healthy Young Men Living in Polluted Areas. Int. J. Mol. Sci..

[29] Lettieri G., Marra F., Moriello C., Prisco M., Notari T., Trifuoggi M., Giarra A., Bosco L., Montano L., Piscopo M. (2020). Molecular Alterations in Spermatozoa of a Family Case Living in the Land of Fires. A First Look at Possible Transgenerational Effects of Pollutants. Int. J. Mol. Sci..

[30] Ishihara K., Warita K., Tanida T., Sugawara T., Kitagawa H., Hoshi N. (2007). Dose Paternal Exposure to 2,3,7,8-Tetrachlorodibenzo-p-Dioxin (TCDD) affect the Sex Ratio of Offspring?. J. Veter. Med. Sci..

[31] Mocarelli P., Gerthoux P.M., Ferrari E., Patterson D.G., Kieszak S.M., Brambilla P., Vincoli N., Signorini S., Tramacere P., Carreri V. (2000). Paternal concentrations of dioxin and sex ratio of offspring. Lancet.

[32] Ryan J.J., Amirova Z., Carrier G. (2002). Sex ratios of children of Russian pesticide producers exposed to dioxin. Environ. Health Perspect..

[33] Anway M.D., Memon M.A., Uzumcu M., Skinner M.K. (2006). Transgenerational Effect of the Endocrine Disruptor Vinclozolin on Male Spermatogenesis. J. Androl..

[34] Kolasa A., Marchlewicz M., Wenda-Różewicka L., Wiszniewska B. (2011). DHT deficiency perturbs the integrity of the rat seminiferous epithelium by disrupting tight and adherens junctions. Folia Histochem. Cytobiol..

[35] Ashby J., Tinwell H., Odum J., Lefevre P. (2004). Natural variability and the influence of concurrent control values on the detection and interpretation of low-dose or weak endocrine toxicities. Environ. Health Perspect..

[36] Gao W., Kearbey J.D., Nair V.A., Chung K., Parlow A.F., Miller D.D., Dalton J.T. (2004). Comparison of the pharmacological effects of a novel selective androgen receptor modulator, the 5alpha-reductase inhibitor finasteride, and the anti-androgen hydroxyl flutamide in intact rats: New approach for benign prostate hyperplasia. Endocrinology.

[37] Clermont Y., Perey B. (1957). Quantitative study of the cell population of the seminiferous tubules in immature rats. Am. J. Anat..

[38] Huang D.W., Sherman B.T., Lempicki R.A. (2009). Systematic and integrative analysis of large gene lists using DAVID bioinformatics resources. Nat. Protoc..

[39] Yoon H., Enquist L., Dulac C. (2005). Olfactory Inputs to Hypothalamic Neurons Controlling Reproduction and Fertility. Cell.

[40] Opatova P., Jennifer Vonk J., Shackelford T. (2017). Peak fertility. Encyclopedia of Animal Cognition and Behaviour.

[41] Spehr M., Schwane K., Riffell J.A., Zimmer R.K., Hatt H. (2006). Odorant receptors and olfactory-like signaling mechanisms in mammalian sperm. Mol. Cell. Endocrinol..

[42] Parmentier M., Libert F., Schurmans S., Schiffmann S., Lefort A., Eggerickx D., Ledent C., Mollereau C., Gérard C., Perret J. (1992). Expression of members of the putative olfactory receptor gene family in mammalian germ cells. Nat. Cell Biol..

[43] Vanderhaeghen P., Schurmans S., Vassart G., Parmentier M. (1993). Olfactory receptors are displayed on dog mature sperm cells. J. Cell Biol..

[44] Zhang X., Rogers M., Tian H., Zou D.-J., Liu J., Ma M., Shepherd G.M., Firestein S.J. (2004). High-throughput microarray detection of olfactory receptor gene expression in the mouse. Proc. Natl. Acad. Sci. USA.

[45] Vanderhaeghen P., Schurmans S., Vassart G., Parmentier M. (1997). Specific Repertoire of Olfactory Receptor Genes in the Male Germ Cells of Several Mammalian Species. Genomics.

[46] Spehr M., Gisselmann G., Poplawski A., Riffell J.A., Wetzel C.H., Zimmer R.K., Hatt H. (2003). Identification of a Testicular Odorant Receptor Mediating Human Sperm Chemotaxis. Science.

[47] Ottaviano G., Zuccarello D., Menegazzo M., Perilli L., Marioni G., Frigo A.C., Staffieri A., Foresta C. (2013). Human olfactory sensitivity for bourgeonal and male infertility: A preliminary investigation. Eur. Arch. Oto-Rhino-Laryngol..

[48] Djahanbakhch O., Ezzati M., Saridogan E., Bahathiq A.O.S., Tan S.L., Ledger W.L. (2010). Physiology and pathophysiology of tubal transport: Ciliary beat and muscular contractility, relevance to tubal infertility, recent research, and future directions. The Fallopian Tube in Infertility and IVF Practice.

[49] Sun F., Bahat A., Gakamsky A., Girsh E., Katz N., Giojalas L., Tur-Kaspa I., Eisenbach M. (2005). Human sperm chemotaxis: Both the oocyte and its surrounding cumulus cells secrete sperm chemoattractants. Hum. Reprod..

[50] Spehr M., Schwane K., Riffell J.A., Barbour J., Zimmer R.K., Neuhaus E., Hatt H. (2004). Particulate Adenylate Cyclase Plays a Key Role in Human Sperm Olfactory Receptor-mediated Chemotaxis. J. Biol. Chem..

[51] Hunter R. (2003). Physiology of the Graafian Follicle and Ovulation.

[52] Olaniyan O.T., Dare A., Okotie G.E., Adetunji C.O., Ibitoye B.O., Eweoya O., Dare J.B., Okoli B.J. (2021). Ovarian odorant-like biomolecules in promoting chemotaxis behavior of spermatozoa olfactory receptors during migration, maturation, and fertilization. Middle East Fertil. Soc. J..

[53] Karteris E., Zervou S., Pang Y., Dong J., Hillhouse E.W., Randeva H.S. (2006). Progesterone signaling in human my-ometrium through two novel membrane G protein-coupled receptors: Potential role in functional progesterone withdrawal at term. Mol. Endocrinol..

[54] Yao Y., Ho P., Yeung W.S. (2000). Effects of human follicular fluid on the capacitation and motility of human spermatozoa. Fertil. Steril..

[55] Ottaviano G., Zuccarello D., Frasson G., Scarpa B., Nardello E., Foresta C. (2013). Olfactory sensitivity and sexual desire in young adult and elderly men: An introductory investigation. Am. J. Rhinol. Allergy.

[56] Ottaviano G., Marioni G., Frasson G., Zuccarello D., Marchese-Ragona R., Staffieri C. (2015). Olfactory threshold for bourgeonal and sexual desire in young adult males. Med. Hypotheses.

[57] Bauman T.M., Sehgal P.D., Johnson K.A., Pier T., Bruskewitz R.C., Ricke W.A., Huang W. (2014). Finasteride treat-ment alters tissue specific androgen receptor expression in prostate tissues. Prostate.

[58] Di Loreto C., La Marra F., Mazzon G., Belgrano E., Trombetta C., Cauci S. (2014). Immunohistochemical Evaluation of Androgen Receptor and Nerve Structure Density in Human Prepuce from Patients with Persistent Sexual Side Effects after Finasteride Use for Androgenetic Alopecia. PLoS ONE.

[59] Cecchin E., De Mattia E., Mazzon G., Cauci S., Trombetta C., Toffoli G. (2014). A Pharmacogenetic Survey of Androgen Receptor (CAG)N and (GGN)N Polymorphisms in Patients Experiencing Long Term Side Effects after Finasteride Discontinuation. Int. J. Biol. Markers.

[60] Cauci S., Chiriacò G., Cecchin E., Toffoli G., Xodo S., Stinco G., Trombetta C. (2017). Androgen Receptor (AR) Gene (CAG)n and (GGN)n Length Polymorphisms and Symptoms in Young Males With Long-Lasting Adverse Effects After Finasteride Use Against Androgenic Alopecia. Sex. Med..

[61] Kur P., Kolasa-Wołosiuk A., Grabowska M., Kram A., Tarnowski M., Baranowska-Bosiacka I., Rzeszotek S., Piasecka M., Wiszniewska B. (2021). The Postnatal Offspring of Finasteride-Treated Male Rats Shows Hyperglycaemia, Elevated Hepatic Glycogen Storage and Altered GLUT2, IR, and AR Expression in the Liver. Int. J. Mol. Sci..

[62] Traish A.M. (2018). The Post-finasteride Syndrome: Clinical Manifestation of Drug-Induced Epigenetics Due to Endocrine Disruption. Curr. Sex. Health Rep..

[63] Bechis S.K., Otsetov A.G., Ge R., Olumi A.F. (2014). Personalized Medicine for the Management of Benign Prostatic Hyperplasia. J. Urol..

[64] Naz R.K., Sellamuthu R. (2006). Receptors in spermatozoa: Are andrology they real?. J. Androl..

[65] O’Hara L., Smith L.B. (2015). Androgen receptor roles in spermatogenesis and infertility. Best Pr. Res. Clin. Endocrinol. Metab..

